# Evaluation of the clinical efficacy of using an inverted triangular cannulated compression screw in combination with positive or negative buttress reduction for the healing of femoral neck fractures

**DOI:** 10.1186/s12891-024-07673-x

**Published:** 2024-07-15

**Authors:** Gang Wang, Cui Tang, Yong Tang, Rui Wang, Tugang Shen, Chundao Xu, Jian Yu, Gaokai Li

**Affiliations:** 1 School of Life and Health, Huzhou College, No. 1, Bachelor Road, Huzhou, 313000 Zhejiang China; 2 Zhejiang Xinda Hospital, No 288, Xinguang Avenue, Huzhou, 313000 Zhejiang China; 3 Department of Orthopedics, 72nd Group Army Hospital of the PLA, No 9, Chezhan Road, Huzhou, 313000 Zhejiang China; 4 Department of Orthopedics, South Taihu Hospital affiliated with Huzhou College, No. 1566, Gangnan Road, Huzhou, 313000 Zhejiang China

**Keywords:** Inverted triangular cannulated compression screw, Positive buttress reduction, Negative buttress reduction, Femoral neck fracture, Clinical efficacy evaluation

## Abstract

**Objective:**

We aimed to compare the clinical efficacy of inverted triangular cannulated compression screws combined with Gotfried positive or negative buttress reduction in the healing of femoral neck fractures.

**Methods:**

Between October 2017 and March 2021, 55 patients with femoral neck fractures underwent treatment using inverted triangular cannulated compression screws combined with Gotfried positive or negative buttress reduction. Among these patients, 29 received inverted triangular cannulated compression screws combined with Gotfried positive buttress reduction treatment. This group consisted of 16 males and 13 females, with an average age of 43.45 ± 8.23 years. Additionally, 26 patients received inverted triangular cannulated compression nails combined with Gotfried negative buttress reduction treatment. This group included 14 males and 12 females, with an average age of 41.96 ± 8.69 years. Postsurgery, various measurements were taken, including the degree of shortening of the femoral neck, degree of bone nonunion, degree of fixation failure, degree of ischemic necrosis of the femoral head, and Harris score of the hip joint.

**Results:**

All patients were followed up for a minimum of 18 months. The group that underwent treatment with an inverted triangular cannulated compression screw combined with Gotfried positive buttress reduction did not experience any cases of bone nonunion, fixation failure, or ischemic necrosis of the femoral head. In the group that received treatment with inverted triangle cannulated compression screws combined with Gotfried negative buttress reduction, there was one case of bone nonunion, three cases of early fixation failure, and one case of ischemic necrosis. Ultimately, five patients (19.23% of the total) underwent joint replacement surgery. The average shortening lengths in the vertical plane were 4.07 ± 1.98 mm and 8.08 ± 3.54 mm, respectively. In the horizontal plane, the average shortening lengths were 3.90 ± 1.57 mm and 7.77 ± 3.31 mm, respectively. At the last follow-up, the group that received Gotfried positive buttress reduction had a greater Harris hip joint score.

**Conclusion:**

The success rate of combining inverted triangular cannulated compression screws with Gotfried positive buttress reduction surgery is relatively high. This surgical approach effectively prevents femoral neck shortening and improves hip joint function. Moreover, it is crucial to avoid negative buttress reduction when managing femoral neck fractures.

## Introduction

Femoral neck fracture is a prevalent hip fracture in clinical practice, accounting for approximately 57% of all hip fractures, and can affect individuals across different age groups [1]. The treatment methods for patients with different age groups and types of fractures vary. For patients younger than 65 years with femoral neck fractures, the preferred treatment methods are closed reduction internal fixation and femoral head preservation [2, 3]. Displacement and inadequate reduction of fractures significantly contribute to nonunion [4, 5].

Achieving anatomic reduction is often considered crucial for promoting fracture healing and preventing complications [6]. However, some femoral neck fractures are difficult to reduce anatomically through closed traction reduction methods, which can damage the residual blood supply and affect fracture healing. Open reduction can also impair the blood supply to the femoral head. Therefore, nonanatomical reduction methods are needed for these difficult-to-reduce fractures.

**Fig. 1:**
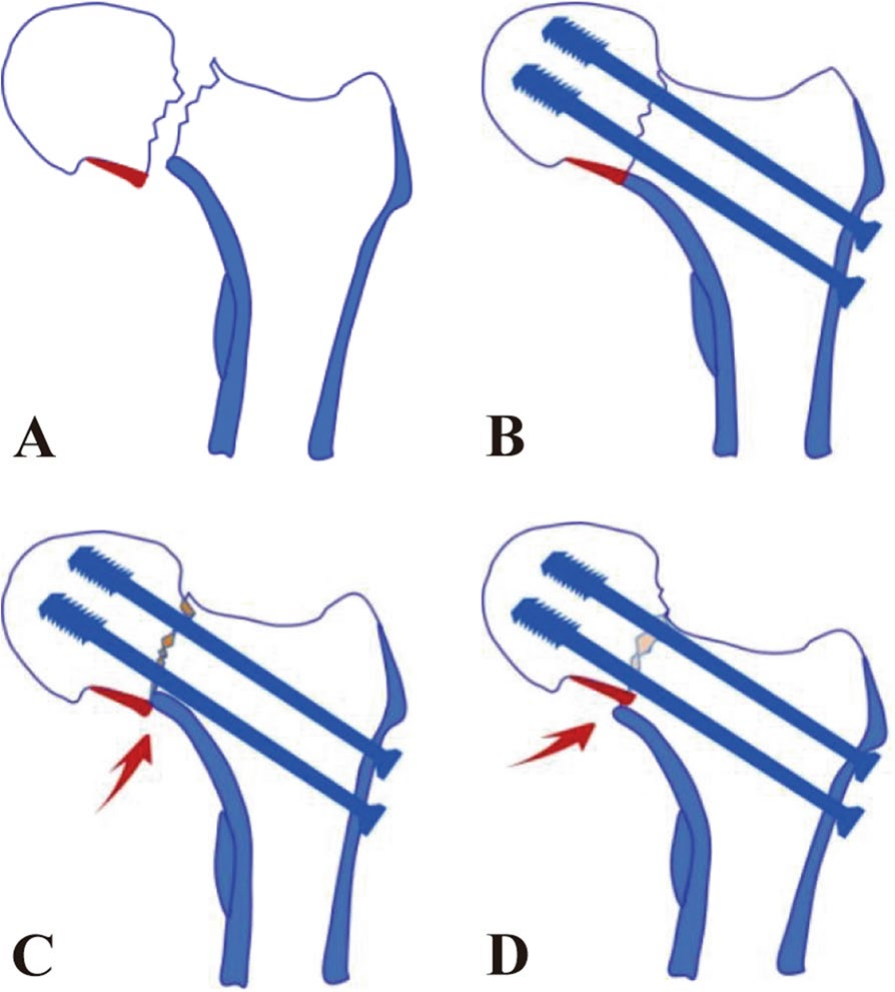
Four modes of femoral neck fractures were described. **A** femoral neck fracture; **B** anatomical reduction of femoral neck fracture; **C** negative buttress reduction of femoral neck fracture; **D** positive buttress reduction of femoral neck fracture

Closed reduction techniques offer several advantages over open reduction, including improved fracture healing rates, faster healing time, and reduced risk of infection [7]. For refractory femoral neck fractures, achieving anatomical reduction is often difficult, and repeated closed traction reduction can also damage the remaining blood supply of the femoral head. However, since 2012, Gotfried et al. [8] proposed the concept of positive buttress reduction for femoral neck fractures, which has also shown good clinical efficacy (Fig. 1). Many studies have compared the clinical efficacy between these two types of bracing and anatomical reduction, but few studies have compared the clinical efficacy of positive bracing and negative bracing, so this deserves further study.

We conducted a retrospective study on patients with femoral neck fractures (Garden III-IV, OTA 31-b2.3 or 31-b3) who have been treated with closed positive or negative support reduction combined with inverted triangular hollow compression screws since October 2017. There have been few reports on the comparative study of these two reduction methods. The fixation method reported by Gotfried et al. [8, 9]used PH screws (physiological hip screws) and PCCPs (percutaneous compression plates), while in our study, we used inverted triangular cannulated compression screws. At present, it is believed that the use of inverted triangular cannulated compression screws is the best fixation method, although complications may still occur, such as femoral neck shortening, bone nonunion, and femoral head necrosis [10–12]. To our knowledge, this is the first attempt to compare the clinical efficacy and complications of the combination of inverted triangular cannulated compression screws and two reduction methods in the treatment of femoral neck fractures.

The aim of this study is to compare the clinical efficacy of closed positive buttress reduction versus closed negative buttress reduction, both combined with inverted triangular hollow compression screws, in the treatment of femoral neck fractures (Garden III-IV, OTA 31-b2.3 or 31-b3) in middle-aged and young adults.

## Materials and methods

### Patients

This retrospective study was conducted at a single orthopedic center. All patients signed an informed consent form before surgery, and the approval of the institutional review board was obtained. Our study period was from October 2017 to March 2021. A total of 55 patients with femoral neck fractures received closed positive buttress reduction and negative buttress reduction combined with cannulated compression screw internal fixation. Based on the different qualities of fracture reduction, we divided patients into two groups: Group I (inverted triangular cannulated compression screw combined with Gotfried positive buttress reduction) and Group II (inverted triangular cannulated compression screw combined with Gotfried negative buttress reduction).

The inclusion criteria for this study were as follows: (1) all fracture types were displaced femoral neck fractures, specifically Garden type III-IV or OTA 31-b2.3 or 31-b3 fractures. (2) All patients underwent closed reduction and were fixed with inverted triangle cannulated compression screws. (3) Reduction quality was assessed based on the standards of positive buttress reduction and negative buttress reduction. (4) The ages of the participants ranged between 20 and 60 years. (5) The follow-up period for all patients was more than 18 months. (6) No patients had medial or posterior comminution; (7) all patients had normal bone density; (8) ‘The Garden index’ was used to evaluate the reduction status of patients between 155° and 180°; and (9) the time interval between injury and surgery in all patients was within 48 hours.

The exclusion criteria for this study were as follows: (1) pathological fracture or old fracture (fracture time > 3 weeks). (2) Patients with severe underlying diseases, including diseases of the immune system and blood diseases. (3) Patients with osteoporosis (T value < -2.5). (4) severe conditions such as systemic multiple fractures or multiple injuries and (5) severe hip osteoarthritis or hip deformity.

This study involved two sample groups of patients. Group I consisted of 29 patients who were treated with inverted triangular cannulated compression screws combined with the Gotfried positive buttress reduction technique. Among them, 16 were male and 13 were female, with an average age of 43.45 ± 8.23 years. Group II included 26 patients who were treated with inverted triangular cannulated compression screws combined with the Gotfried negative buttress reduction technique. Among them, 14 were male and 12 were female, with an average age of 41.96 ± 8.69 years. Gotfried positive buttress refers to the distal lower edge of a femoral neck fracture, located on the inner side of the proximal lower edge of the fracture. On the other hand, negative buttress reduction refers to the opposite direction of displacement (Fig. 1). We analyzed independent variables such as the follow-up period (month), Garden's alignment index, Harris hip score, femoral neck shortening in the vertical plane, femoral neck shortening in the horizontal plane, early failure of fixation (%), AVN (%), and conversion to prosthetic replacement. Among these variables, sex, age (years), BMI, cause of injury, Garden classification, OTA classification, and injury to preoperative time (hours) may be potential confounding variables.

Table 1 presents the general preoperative information. Both groups of patients underwent surgery performed by the same team of skilled orthopedic surgeons. The postoperative Harris score of the hip joint, along with the occurrence of complications (such as nonunion, failure of early fixation, and ischemic necrosis of the femoral head), and femoral neck shortening were compared between the two groups.

**Table 1:** Preoperative clinical data

	Group I	Group II	χ^2^/t value	*P* value
Gender				
Male	16(55.17%)	14(53.85%)	0.0097	0.9214
Female	13(44.83%)	12(46.15%)		
Age(years)	43.45±8.23	41.96±8.69	0.651	0.518
BMI	21.21±2.64	20.85±2.81	0.491	0.625
Causes of injuries				
Traffic accidents	19(65.52%)	17(65.38%)	0.0001	0.9918
Sport injuries	10(34.48%)	9(34.62%)		
Garden classification				
Type III	21(72.41%)	19(73.08%)	0.003	0.956
Type IV	8(27.59%)	7(26.92%)		
OTA classification				
OTA 31-B2.3	10	7	0.2535	0.8353
OTA 31-B3.1	7	8	0.2210	
OTA 31-B3.2	7	5	0.1513	
OTA 31-B3.3	5	6	0.2334	
Injury to preoperative time (hours)	38.93±7.20	39.88±5.78	-0.538	0.593

### Treatment protocol

**Fig. 2:**
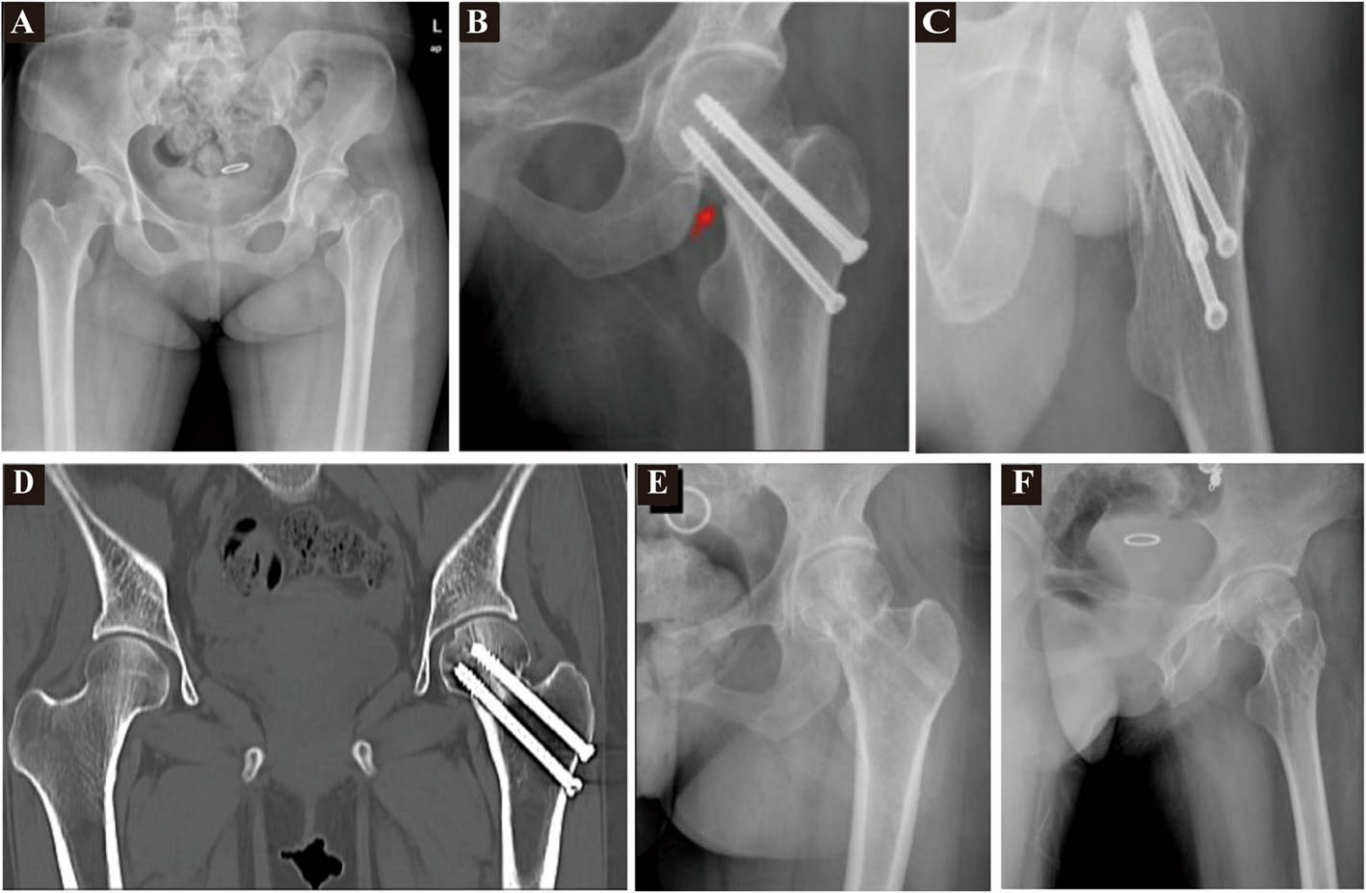
A 55-year-old female with a femoral neck fracture. **A** femoral neck fracture double hip positive-position X-ray; **B** and **C** postoperative and lateral X-ray of femoral neck fracture (positive support at the red arrow); **D** 1 year after the operation of femoral neck fracture, double hip CT showed complete bone healing of the fracture; **E** and **F**: 18 months after surgery, the femoral neck fracture was treated with internal fixation and the positive and lateral radiographs were removed. The fracture was completely fixed and healed, and no obvious necrosis and collapse of the femoral head was observed

**Fig. 3:**
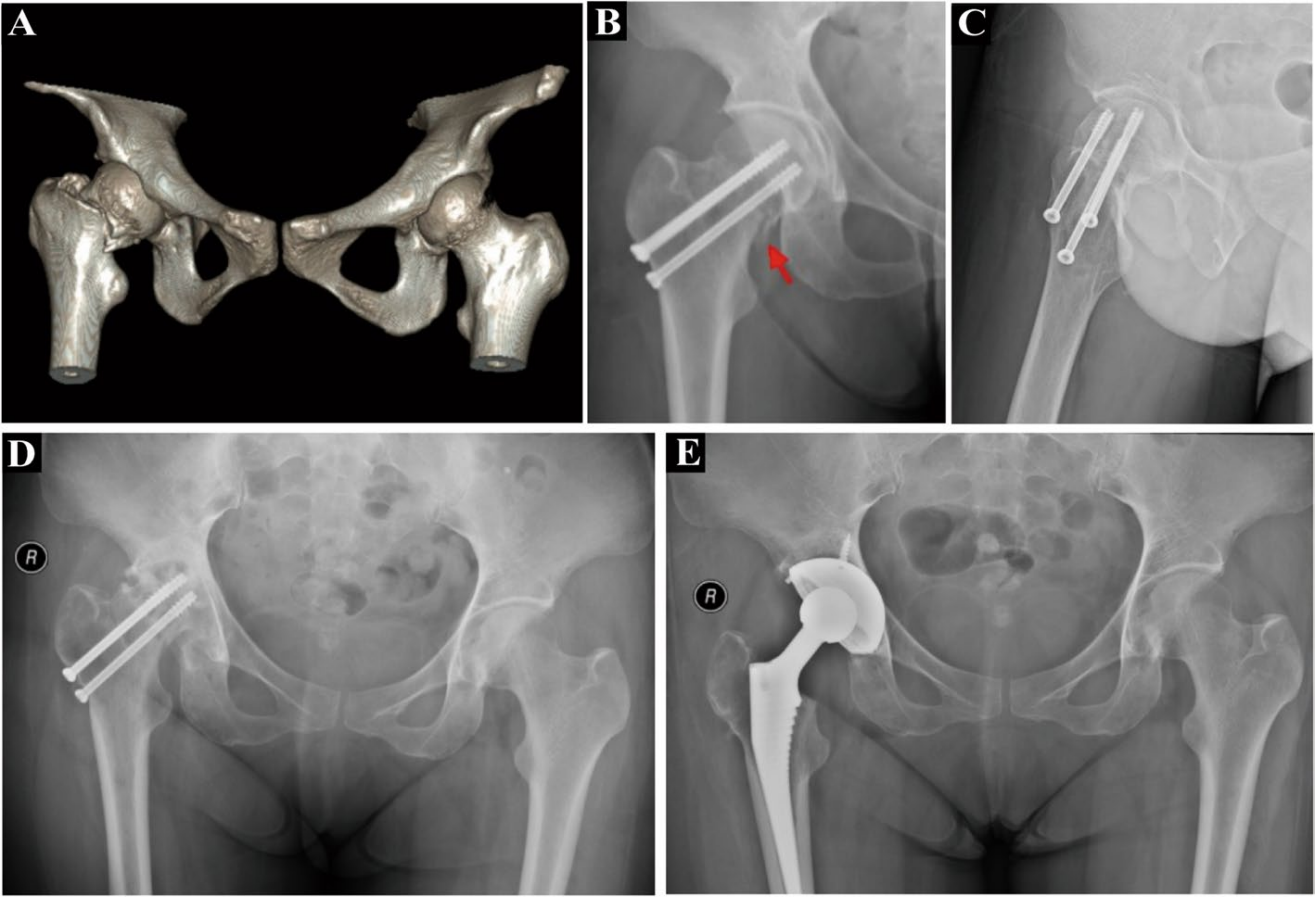
A 48-year-old female patient with femoral neck fracture. **A** CT scan of femoral neck fracture and double hip; **B** and **C** postoperative and lateral X-ray of femoral neck fracture (negative support at the red arrow); **D** X film 18 months after surgery for femoral neck fracture, femoral head has collapsed and become necrotic; **E** X-ray after right total hip replacement 18 months after surgery

All patients underwent oral intubation anesthesia and were placed in a recumbent position on a surgical traction bed with a hip pad elevated at an angle of 10 to 15 degrees. Closed traction reduction technology was used, starting with an outreach extorsion limb of approximately 20 degrees, followed by a spin limb rotation within the range of 20 to 30 degrees. The quality of femoral neck reduction was determined using a C-arm X-ray machine through anteroposterior and lateral fluoroscopy. Once fracture reduction was achieved, the fracture was fixed using three cannulated screws of an inverted triangle type. Initially, a Kirschner wire was inserted through the anterior neck of the femur, guiding the placement of three needles from the femoral lateral cortex. The needles were advanced at a forward angle of 10 degrees to enter three cannulated compression screws. The goal was to position the three cannulated screws in the middle and lower quadrants of the femoral neck and femoral head, with the screw tip approximately 5 to 10 mm away from the subchondral bone. Finally, C-arm fluoroscopy was performed again to confirm the fracture reduction and screw position. Two reduction methods were employed: positive buttress reduction (Fig. 2) and negative buttress reduction (Fig. 3).

**Fig. 4:**
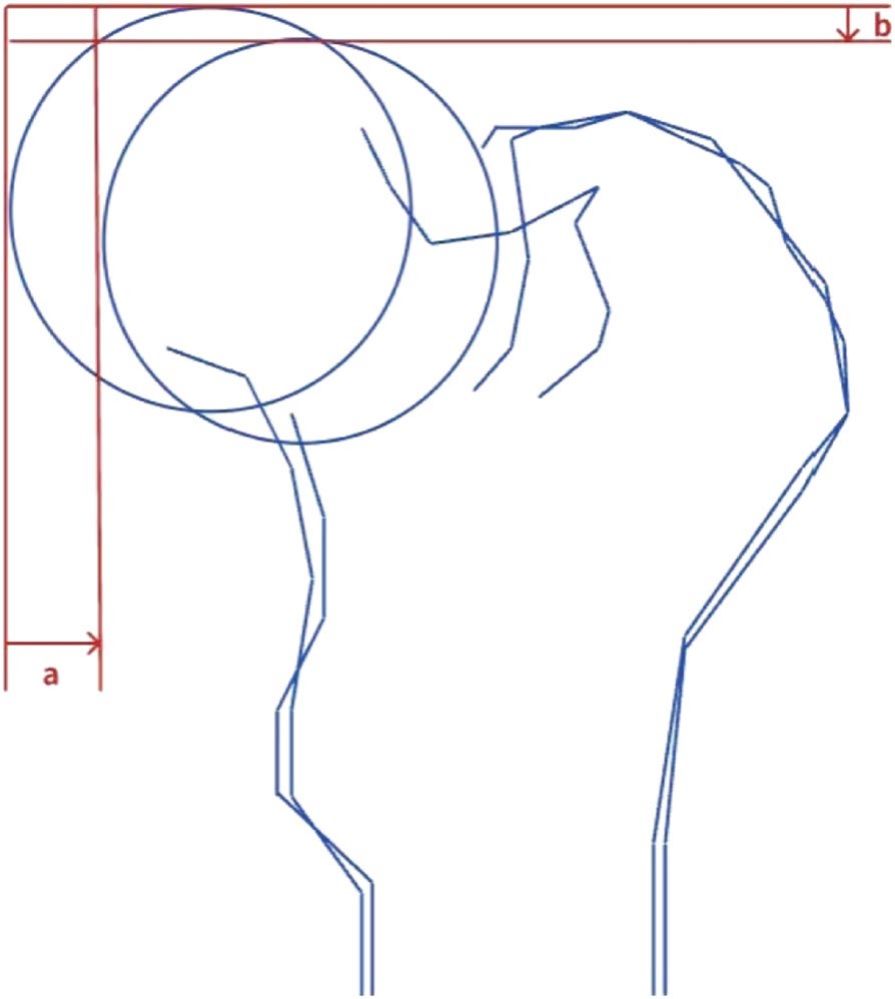
Femoral head displacement change measurement method

All patients received comprehensive treatment, which included anti-infection, anticoagulation, rehydration, and anti-rotation shoe fixation. The patients were discharged once their condition stabilized. Follow-up visits were conducted in the outpatient field, with patients being reviewed monthly before fracture healing and every six months after healing. Three months after surgery, partial weight-bearing exercise was gradually introduced based on the fracture healing status observed in the radiographs. Crutches and complete weight-bearing activities were determined 4 to 6 months after surgery, depending on the fracture healing status. During the 18-month follow-up after surgery, pain and limited movement were observed. The recovery of hip joint function was evaluated using the Harris score. After 18 months of follow-up, the method described in the literature [13, 14] was adopted to measure the shortening of the femoral neck using Adobe Photoshop CS6 with digital X-rays. As shown in Fig. 4, after superimposing the anteroposterior X-ray images of the patient's healthy and affected hip joints, the changes in the displacement of the femoral heads were compared. The vector sum of the horizontal (a) and vertical (b) displacements of the femoral head is considered the length of neck shortening. The correction was made using the ratio of the actual length of the screw diameter to the length on the X-ray film. Measurements were carried out multiple times by three experienced senior physicians, and the average value was taken. The degree of femoral neck shortening was evaluated in both the horizontal and vertical directions, and the number of patients with femoral neck shortening greater than 5 mm was recorded for comparison.

Radiological assessment: Postoperative X-ray films were utilized to evaluate the quality of fracture reduction. The method for evaluating the buttress reduction technique was also assessed by three experienced senior physicians, who used the hospital's His imaging system to perform proportional enlargement for evaluation, resulting in high reliability. The anteroposterior and lateral alignment angles of each patient were measured based on the Garden alignment index [15]. Early fixation failure was defined as the need for reoperation within 6 months due to fixation failure. Nonunion was defined as the presence of a visible fracture line 12 months after surgery.

### Statistical analysis

Statistical analysis was conducted using SPSS 23.0 statistical software (SPSS Inc., Chicago, IL, USA). The measurement data are presented as the means ± standard deviations. A t test was used to compare two independent samples. The comparison of counting data was performed using the chi-square test or exact probability method. A statistically significant result was considered when *P* < 0.05. The number of patients who converted to joint replacement, including those with bone nonunion, early fixation failure, and ischemic necrosis of the femoral head, was recorded.

## Results

Table 1 presents the fundamental data of the two patient groups prior to surgery. There were no significant differences observed between the two groups in terms of sex, age, body mass index (BMI), surgical reasons, fracture Garden classification, OTA classification, or the time elapsed between injury and surgery (*P*>0.05).

The postoperative outcomes of the two groups are shown in Table 2. In Group I, the follow-up time was 22.5±3.19 months. The garden correlative index showed an anteroposterior position of 162.52±2.29° and a lateral position of 177.69±1.82°. The Harris scores were 77.62±2.93, 87.69±3.08, and 93.48±3.03 at 3, 6, and 18 months after surgery, respectively. The average magnitude of shortening of the femoral neck was 4.07±1.98 mm in the vertical plane and 3.90±1.57 mm in the horizontal plane. There were 9 patients (31.03%) and 10 patients (34.48%) with femoral neck shortening greater than 5 mm in the vertical plane and horizontal plane, respectively. In this group, there were no cases of bone nonunion, failure of early fixation, or ischemic necrosis of the femoral head.

**Table 2:** Postoperative results

	Group I	Group II	χ^2^/t value	*P* value±
Follow-up period (month)	22.55±3.19	22.42±3.05	0.152	0.879
Garden’s alignment index				
Anteroposterior (degree)	162.52±2.29	162.46±2.76	0.082	0.935
Lateral (degree)	177.69±1.82	177.58±1.72	0.235	0.815
Harris Hip Score				
3 months after surgery	77.62±2.93	76.69±2.26	1.322	0.198
6months after surgery	87.69±3.08	85.04±3.42	3.022	0.004**
18 months after surgery	93.48±3.03	89.23±3.58	4.768	0.000**
Femoral neck shortening				
In the vertical plane				
Mean decrease (mm)	4.07±1.98	8.08±3.54	-5.098	0.000**
<5mm	20(68.97%)	9(34.62%)	6.490	0.011*
>5mm	9(31.03%)	17(65.38%)		
In the horizontal plane				
Mean decrease (mm)	3.90±1.57	7.77±3.31	-5.438	0.000*
<5mm	19(65.52%)	9(34.62%)	5.238	0.022*
>5mm	10(34.48%)	17(65.38%)		
Nonunion (%)	0	1(3.85%)		0.473
Early failure of fixation (%)	0	3(11.54%)		0.099
AVN(%)	0	1(3.85%)		0.473
Conversion to prosthetic replacement	0	5(19.23%)		0.019*

^*^P<0.05; **P<0.01

In Group II, the follow-up period was 22.42±3.05 months. The Garden index for the AP position was 162.46±2.76°, and that for the LP position was 177.58±1.72°. The Harris scores were 76.69±2.26, 85.04±3.42, and 89.23±3.58 at 3, 6, and 18 months after surgery, respectively. The average magnitude of shortening of the femoral neck was 8.08±3.54 mm in the vertical plane and 7.77±3.31 mm in the horizontal plane. Seventeen patients (65.38%) had femoral neck shortening greater than 5 mm in both the vertical and horizontal planes. In this group, there was 1 case of bone nonunion (*POWELL II*), 3 cases (2 cases *POWELL II*, 1 case *POWELL III*) of early fixation failure due to implant loosening, and 1 case of ischemic necrosis of the femoral head (*POWELL II*). Five patients (19.23%) underwent joint replacement surgery.

During the follow-up period, there were statistically significant differences in the Harris score at 6 months (*P*=0.004) and 18 months (*P*=0.000) between the two groups. The femoral neck also showed statistically significant differences in vertical and horizontal shortening (*P*=0.000). Moreover, there were statistically significant differences between the two groups in terms of femoral neck shortening > 5 mm in both the vertical plane (*P*=0.0109) and the horizontal plane (*P*=0.0221). In group II, a total of five patients (19.23%) underwent joint replacement, which was found to be a statistically significant difference (*P*=0.019).

## Discussion

The optimal surgical method for treating femoral neck fractures is closed anatomic reduction and rigid internal fixation. Various reduction methods are available for fracture reduction [16–18]. However, achieving anatomic reduction may be difficult in some patients with displaced femoral neck fractures. Treating femoral neck fractures with nonanatomic reduction remains a challenge.

Recently, a study compared the effect of positive and negative buttress reduction on the healing of femoral neck fractures [19]. The clinical efficacy of two nonanatomic reduction methods for treating these fractures was compared. The positive buttress reduction group had no cases of early fixation failure, whereas the negative buttress reduction group had 3 cases. Both groups underwent inverted triangle configuration cannulated screw fixation, but the positive buttress group had a lower failure rate due to the strong buttress of the medial femoral cortex. The positive buttress reduction group had no cases of bone nonunion or avascular necrosis of the femoral head. In the negative buttress group, there was 1 case of bone nonunion and 1 case of ischemic necrosis of the femoral head, resulting in 5 patients needing joint replacement surgery. In the process of healing a femoral neck fracture, it is crucial to prioritize the protection of the blood supply to the femoral head. Additionally, we should focus on ensuring the biomechanical stability of the femoral head. Zhang et al. [20] conducted an analysis on the biomechanical mechanism buttressed by Gotfried. During fracture healing, the three cannulated screws provide sliding compression, causing the fracture line to absorb and generate stress concentrated at the bone-screw interface. However, increasing interfacial pressure can increase the risk of varus displacement of the femoral head. In the event of femoral head varus tendency, positive buttress reduction can facilitate contact between the internal cortex and the cortex of the femoral neck. This contact effectively resists longitudinal shear stress between the fracture blocks and prevents further varus displacement of the femoral head. The negative buttress reduction technique does not effectively prevent the risk of femoral head varus cutting and increases the possibility of cortical contact with cancellous bone. Previous literature [21] has indicated that varus displacement is a significant predictor of failure in fixing femoral neck fractures. In our study, the positive buttress group did not experience varus deformity or bone nonunion, whereas the negative buttress group had 3 cases of varus deformity and 1 case of bone nonunion after fixation failure. Therefore, the positive buttress reduction technique offers a mechanically and biomechanically stable environment for the healing of femoral neck fractures.

Another key issue in the healing process of femoral neck fractures is neck shortening. It is agreed that femoral neck shortening after cannulation of compression screws for femoral neck fractures is common [22]. Due to the shortening of one's femoral neck, the tension distribution between the implant and the fracture site can be uneven, which can lead to an increased likelihood of an implant sliding or even breaking [23]. In addition, shortening of the femoral neck results in a reduction in the eccentricity of the femoral neck and weakening of the abductor arm and affects gait speed, symmetry, physical function, and hip joint dysfunction [24]. Zlowodzki et al. [14] followed up patients with femoral neck shortening by using the EuroQol questionnaire score and the SF-36 physical function score. The results showed that patients with shortening >5 cm had varying degrees of claudication. In our study, positive and negative buttress reduction techniques were used to treat displaced femoral neck fractures in adults, and previously reported methods [13, 14] were used to quantitatively and qualitatively assess the incidence of femoral neck shortening. Our study revealed that the average shortening in the vertical plane of the positive buttress group was 4.07±1.98 mm, and the average shortening in the horizontal plane was 3.90±1.57 mm. In the positive buttress reduction group, there were 9 patients (31.03%) and 10 patients (34.48%) with femoral neck shortening greater than 5 mm in the vertical and horizontal planes, respectively. However, in the negative buttress reduction group, the average magnitude of shortening in the vertical plane was 8.08±3.54 mm, and the average magnitude of shortening in the horizontal plane was 7.77±3.31 mm. In the positive buttress reduction group, 17 patients (65.38%) had femoral neck shortening greater than 5 mm in the vertical or horizontal plane. This means that positive buttress reduction can significantly reduce the incidence and degree of severe shortening after femoral neck fracture surgery. The main reason may be that the thickened medial cortex of the femoral neck can effectively buttress the proximal fracture block. In addition, during the healing process, the fracture end can undergo inversion, which can effectively resist shear stress. Thus, positive buttress reduction can reduce femoral neck shortening in the horizontal plane and vertical plane. In this study, the Harris scores of the hip joints in the positive buttress reduction group (87.69±3.08, 93.48±3.03) were significantly better than those in the negative buttress reduction group (85.04±3.42, 89.23±3.58) at the 6-month and 18-month follow-ups. The main reason for patients' improvements in functional and pain relief may be that positive buttress reduction can effectively prevent femoral neck shortening.

The stability of the medial buttress for femoral neck fractures is important for fracture healing and prevention of femoral neck shortening. In particular, for Pauwels III femoral neck fractures, the medial buttress screw can effectively resist the vertical shear force at the end of the fracture, promote the stability and healing of the fracture end, and reduce the occurrence of postoperative complications [25]. Ye Y al. [26] treated vertical femoral neck fractures with open reduction with a medial buttress plate combined with three cannulated screws. A medial buttress plate can enhance the stability of femoral neck fracture fixation. However, at the same time, the loss of the sliding compression effect of three cannulated screws is not conducive to fracture healing. At the same time, the residual femoral head blood supply was lost by open reduction. This method has also increased the operation time and the operation difficulty. Compared with medial buttress plate technology, positive buttress reduction technology has obvious advantages. First, it is achieved through closed reduction and does not damage the blood supply, and the operation is relatively simple and easy. Second, positive buttress reduction not only results in medial femoral cortical buttress but also retains the sliding compression effect of the cannulated screw in the process of fracture healing.

We are aware of the limitations of this study. First, this was a single-center study with a small sample size. Second, the follow-up time was relatively short. For some AVN (vascular necrosis) patients, longer follow-up statistics are needed. The lack of AVN patients in group I in this study may be due to the short follow-up time. Finally, this was a retrospective study, and there may be some memory bias in the follow-up. Therefore, larger samples and long-term follow-up studies are needed to further confirm the advantages of the positive buttress reduction technique.

## Conclusion

A positive buttress reduction technique for displaced femoral neck fractures is associated with a high rate of fracture healing and can significantly prevent femoral neck shortening and improve hip joint function. Therefore, in the closed reduction of displaced femoral neck fractures, positive buttress reduction is acceptable, while negative buttress reduction should be avoided as much as possible.

## Data Availability

The results of this study were obtained from the South Taihu Hospital affiliated with Huzhou College. The data analyzed during the present study are all available from the corresponding author upon reasonable request.
